# Rhinology: A Text-Book of Diseases of the Nose and the Nasal Accessory Sinuses

**Published:** 1910-03

**Authors:** 


**Rhinology: A text-book of Diseases of the Nose and the Nasal Accessory Sinuses.** By PATRICK WATSON WILLIAMS, M.D. (Lond). Pp. xvi, 273. London: Longmans, Green and Co. 1910.

It was formerly customary to include rhinology as a small and unimportant addition to a text-book of laryngology, but the subject has made such advances of late that it well deserves a separate consideration, and we have before us a handsome volume of nearly 300 pages, illustrated with 28 stereoscopic plates, 19 ordinary plates and 146 other illustrations, all devoted to the once despised subject of nasal surgery.

The stereoscopic plates are a great feature of the book. Some of the most striking are taken from specimens of Professor Zückerkandl, of Vienna, but many others are from dissections and preparations made by the author himself, and when looked at through the small and handy stereoscope supplied with each copy of the book give a most realistic idea of the somewhat complicated relations of the cells and sinuses which surround the nasal cavities.

Some of the plates are by Professor Onodi, of Budapest, and these show in a most clear manner the relations of the nasal sinuses to the orbit, while many other illustrations render the book invaluable to the student in following the written description of operations on the parts in and around the nose.

The first section is devoted to examination of the nasal passages, which is made easy for the student to follow by reason of the remarkably clear illustrations. After a brief section on the anatomy and physiology of the nose, the subject of rhinitis is dealt with, and the various varieties of this affection described. The author looks upon atrophic rhinitis as a chronic inflammatory degeneration of the nasal mucous membrane due to infection by various organisms, and in some cases determined by conditions of the nature of a tropho-neurosis. He very rightly points out that local treatment is at the best only palliative.

In removal of the hypertrophied pharyngeal tonsil (adenoids) the author advises the use of Gottstein's curette as modified by St. Clair Thomson or Beckmann, and he always holds the curette as one holds a table fork. When both tonsils and adenoid growths have to be removed he first operates on the pharyngeal growths, and then on the tonsils, unless the latter are so large as to interfere with the passage of instruments into the naso-pharynx, his reason being that hemorrhage in the naso-pharynx can be controlled by a plug, so that when the tonsils come to be removed the parts are not encumbered with blood. We think that most operators prefer the opposite order of procedure, as the bleeding from the cut tonsils interferes very little with the passage of the curette into the naso-pharynx.

The section devoted to diseases of the nasal septum is one of the best in the book, and the modern operations on septal deflections are fully described. The author's own operation seems an excellent one. The first makes a small incision on the concave side, through which he elevates the soft parts and afterwards removes the deflected part of the septum through an incision on the convex side. The results of this operation seem all that can be desired, and no one would now wish to go back to the old, clumsy method of crushing the deflected septum with forceps—one blade in each nostril—which some of us used to practise.

The most important sections of the book are naturally those devoted to the diseases and operations of the accessory sinuses. This department of surgery has all sprung up during the last few years, and although it has doubtless been much overdone by some enthusiastic operators, yet has furnished some of the most brilliant advances of modern surgery. The author points out that the large majority of cases of sinusitis are due to influenza, and it is only since the occurrence of influenza epidemics in 1889 that inflammatory diseases of the accessory sinuses have become common affections. When they do occur they give rise to the most distressing symptoms; indeed, the author met with a case where frontal sinus suppuration was the cause of loss of memory in a lady, so that she stole a valuable ring from a friend's house!

We do not doubt that most cases of sinus inflammation yield to general treatment, but here and there cases occur demanding radical procedures, and then the operations described and figured by the author in this book are urgently demanded.

In chronic suppuration of a frontal sinus the mucous membrane is generally diseased beyond the possibility of recovery, and nothing short of obliteration of the sinus will then cure the patient. In order to effect this an operation of some magnitude is necessary, but the author has never had a death after his operation, which doubtless looks more formidable than it really is, and it is surprising how little deformity is left afterwards, as is proved by photographs given in this book of patients after their recovery.

We consider that this work by our Bristol *confrère* will meet a much-felt want, and will reflect honour on Bristol surgery.

The author's former work on the nose and throat, which ran through four editions, has been out of print for some years; we therefore look forward to the corresponding volume on Laryngology, shortly to appear as a companion to this one, which is an ideal text-book on diseases of the nose, and cannot fail to be of great service to the general practitioner as well as to the operating surgeon.

Considering that it includes such an excellent atlas of plates, it is extraordinary value at the published price, and we may congratulate the publishers on their enterprise.

